# Administration of bovine casein-derived peptide prevents cognitive decline in Alzheimer disease model mice

**DOI:** 10.1371/journal.pone.0171515

**Published:** 2017-02-03

**Authors:** Li-Juan Min, Yodai Kobayashi, Masaki Mogi, Kana Tsukuda, Akio Yamada, Koji Yamauchi, Fumiaki Abe, Jun Iwanami, Jin-Zhong Xiao, Masatsugu Horiuchi

**Affiliations:** 1 Department of Molecular Cardiovascular Biology and Pharmacology, Ehime University, Graduate School of Medicine, Shitsukawa, Tohon, Ehime, Japan; 2 Morinaga Milk Industry Co., Ltd., Zama, Kanagawa, Japan; Hungarian Academy of Sciences, HUNGARY

## Abstract

There is a growing interest in identifying natural food ingredients that may serve to prevent dementia such as that due to Alzheimer disease (AD). Peptides derived from food proteins have been demonstrated to have various physiological activities such as a hypotensive action. Recent findings have indicated possible associations of hypertension with AD progression, and suggest that angiotensin converting enzyme (ACE) inhibitors with potential to pass through the blood brain barrier (BBB) may reduce the risk of AD. In this study, we investigated the effect of milk peptide (CH-3) on cognitive function in AD model mice. CH-3 contains a tripeptide (methionine-lysine-proline, MKP) that has been found to have a strong ACE inhibitory effect and the potential to pass through the BBB. Adult male ddY mice were used in this study, and an animal model of AD was induced by intracerebroventricular (ICV) injection of Aβ1–42. CH-3 (250 mg/kg/day) or MKP (0.5 mg/kg/day) was orally administered every day starting 2 days before ICV injection. At 3 weeks after ICV injection, cognitive function was evaluated by the Morris water maze test. Brain samples were obtained after behavioral testing, and expression of inflammatory cytokines and NADPH oxidase subunits was measured by real-time quantitative RT-PCR. ICV injection of Aβ1–42 significantly impaired cognitive function compared with that in PBS-injected mice. Daily administration of CH-3 markedly attenuated this Aβ1-42-induced cognitive decline. Aβ1–42 injection significantly enhanced the expression of tumor necrosis factor-α (TNF-α), inducible nitric oxide synthase (iNOS) and p22^phox^ in the mouse hippocampus compared with PBS injection, and showed a tendency to increase the expression of monocyte chemoattractant protein-1 (MCP-1), p47^phox^ and gp91^phox^, whereas CH-3 treatment markedly reduced Aβ1-42-induced TNF-α, MCP-1, iNOS, p47^phox^ and gp91^phox^ expression. Finally, administration of MKP also attenuated Aβ1-42-induced cognitive impairment with an increase in cerebral blood flow. The present study demonstrated that repeated oral administration of CH-3 to AD model mice not only improved cognitive function but also suppressed the expression of inflammatory cytokines and production of oxidative stress, and suggests its therapeutic potential for preventing cognitive impairment in AD.

## Introduction

Alzheimer disease (AD), an irreversible progressive neurodegenerative disorder, is one of the most prevalent neurodegenerative diseases in aging societies. It is associated with memory loss, and its typical symptoms are memory impairment and cognitive decline. It is becoming an increasing burden on not only patients but also their families. In the brain of AD patients, Aβ deposition is a crucial pathological event [1]. Aβ deposition in the brain has been suggested to cause oxidative damage and neuroinflammation, which are closely associated with progression of AD [2–4]. Once the disease becomes clinically obvious, neuronal loss might be too advanced for treatment, and it is becoming more important to prevent the onset of AD through improving lifestyle or diet. Therefore, there is growing interest in identifying possible natural food ingredients that can prevent AD onset and progression.

Many kinds of peptides derived from foods have been reported to have various physiological effects such as a hypotensive action [5,6]. Due to their beneficial health and safety properties, bioactive peptides derived from milk proteins have been well studied by many researchers [7]. We previously reported that bovine casein hydrolysate which is produced by 3 enzymes (“CH-3”) has an antihypertensive effect in vitro and in vivo [8,9]. CH-3 shows angiotensin-converting enzyme (ACE) inhibitory activity, and contains the tripeptide Met-Lys-Pro (MKP) which is the strongest ACE inhibitory peptide found in foods. MKP accounts for more than 30% of the ACE-inhibitory activity of CH-3 and plays a major role in the antihypertensive activity of CH-3 [9].

Recent findings have indicated an association between the brain renin-angiotensin system (RAS) and AD [10]. Angiotensin II (Ang II) is the principal substance of RAS and has a variety of physiological functions [11]. Ang II is generated by ACE, and interestingly the activity of ACE is elevated in the brain of AD patients [12]. The up-regulation of ACE activity may lead to an increase in Ang II level [12]. Brain Ang II is reported to induce reactive oxygen species through NADPH oxidase, and subsequently many transcription factors important for inflammation are activated [13]. Ang II may impair cognitive performance, lower acetylcholine release, and also block the induction of long-term potentiation (LTP) [14]. Some studies have shown beneficial effects of RAS inhibitors on cognitive decline both in AD model animals and AD patients [15–19]. Furthermore, many researchers have suggested that some ACE inhibitors and Ang II receptor blockers (ARBs) have a beneficial effect to prevent cognitive decline in AD model mice [15,17,19]. Ohrui et al. reported that long-term use of a centrally active ACE inhibitor slowed the rate of cognitive decline in patients with mild to moderate AD, and could have a protective effect against the development of AD [16]. Kehoe et al. [10] discussed brain RAS blockade as a possible new treatment option for AD disease.

As mentioned above, the milk peptide CH-3 has an ACE inhibitory effect, and an ACE inhibitor could have a protective effect against the development of AD. In this study, we investigated the possibility that administration of CH-3 could have a preventive effect on cognitive decline in a mouse model of AD. We also examined its potential effects on neuroinflammation and oxidative stress, focusing on the tripeptide MKP, which has a strong ACE inhibitory property.

## Materials and methods

All procedures were performed in accordance with the National Institutes of Health guidelines for the use of experimental animals. The experimental protocol was reviewed and approved by the Animal Studies Committee of Ehime University.

### Animals and treatment

Male 10-week-old ddY mice (SLC, Inc., Japan) were used in this study. Male 10-week-old SHRs (SHR/Hos, SPF) were purchased from Hoshino Laboratory Animals, Inc. (Ibaraki, Japan). These animals were housed in a room in which lighting was controlled (12 hours on and 12 hours off) and room temperature was kept at 25°C. ddY mice were given a MF diet (Oriental Yeast Co., Ltd., Tokyo, Japan) and water ad libitum. SHR rats were fed a MR stock diet (Nihon Nosan Kogyo Co., Ltd., Kanagawa, Japan) and had continuous access to tap water. CH-3 was prepared and provided by Morinaga Milk Industry Co., Ltd. as described previously [9]. Briefly, hydrolysis of lactic casein (Fonterra Co., Ltd., Auckland, New Zealand) was conducted by incubation for 8 hours at 50°C with three different enzymes: Bioprase (EC 3.4.21.62, accepted name: subtilisin, Nagase ChemteX Co., Ltd., Osaka, Japan), Protease N ‘‘Amano” (EC 3.4.24.28, accepted name: bacillolysin, Amano Enzyme Inc., Nagoya, Japan), and PTN6.0S (EC 3.4.4.4, Pancreatic Trypsin Novo, Novozymes Japan Co., Ltd., Chiba, Japan). The reaction was stopped by inactivating the enzymes at 80°C for 6 min. The hydrolysates were spray dried and used for further study. MKP content of CH-3 is 0.045%. The composition of CH-3 is shown in Table 1. Met-Lys-Pro (MKP) was prepared by solid-phase synthesis by Shimadzu Scientific Research Inc. (Kyoto, Japan). CH-3 (250 mg/kg/day) or MKP (0.5 mg/kg/day) was orally administered every day starting 2 days before Aβ1–42 injection.

**Table 1:** Composition of CH-3.

Component	Composition of CH (g/100g)
**Protein**	85.0
**Fat**	0.0
**Carbohydrate**	4.0
**Ash**	7.0
**Moisture**	4.0
**Total**	100

### Intracerebroventricular injection of Aβ1–42

Intracerebroventricular (ICV) injection was performed as described previously with some modification [19]. Briefly, each mouse was fixed in a stereotactic frame, anesthetized with Nembutal in saline, and a 28-gauge needle was inserted unilaterally 1 mm to the right of the midline, 0.2 mm posterior to the bregma and 2.5 mm deep to the skull surface. Aβ1–42 (Peptide Institute, Osaka, Japan) dissolved in PBS was injected intracerebroventricularly at 200 pmol in 3 μl PBS at rate of 1 μl/min using a syringe pump. After the injection, the needle was held in the original location for an additional 3 minutes and then withdrawn. For vehicle control mice, 3 μl PBS was injected. The body temperature was maintained with a heat lamp throughout the procedure and recovery. After they were completely alert, mice were returned to their home room, and normal food and water were given. General locomotor activity and diet volume of mice were checked daily and not changed significantly among all groups until sample preparation. Mice were weighed before and one week after ICV injection, and immediately before each analysis, and showed no significant difference at each time point in each group. In addition, we did not observe a significant change in systolic blood pressure among all groups during reagent administration.

### Morris eater maze test

Spatial learning as a measure of cognitive function of mice at 3 weeks after injection of Aβ1–42 or PBS was evaluated by the Morris water maze test as described previously [19,20]. In brief, each mouse was trained 5 times a day at 20-minute intervals for 5 consecutive days. The test was performed blindly. In each trial, mice were given 120 seconds to find the platform. Swimming was video tracked (AnyMaze, Wood Dale, IL, USA), and latency, path length, swim speed, and cumulative distance from the platform were recorded. Mean swim latency for all of the trials on each day in each group was calculated.

### Cerebral blood flow

After the Morris water maze test, cerebral blood flow (CBF) was determined by laser speckle flowmetry (Omegazone, laser speckle blood flow imager, Omegawave, Tokyo, Japan) as described previously [21]. Mice were anesthetized with Nembutal in saline, and a midline incision was made in the scalp. Anesthesia did not significantly modify blood pressure. The skull was exposed and wet with saline. A 780-nm laser semiconductor laser illuminated the whole skull surface. Mean CBF was measured on the skull surface. Light intensity was accumulated in a charge-coupled device camera and transferred to a computer for analysis. Image pixels were analyzed to produce average perfusion values.

### Real-time quantitative RT-PCR

After CBF measurement, the mouse brain was removed after cardiac perfusion with ice-cold saline. The hippocampus were taken out and frozen in liquid nitrogen and stored at -80°C until use. Total RNA was extracted from the hippocampus with Sepasol reagent (Nacalai Tesque, Inc., Kyoto, Japan). Real-time quantitative RT-PCR was performed using SYBR Premix Ex Taq (Takara Bio Inc., Japan). The level of target gene expression was normalized against expression of a housekeeping gene, glyceraldehyde-3-phosphate dehydrogenase (GADPH), in each sample. PCR primers were as follows: tumor necrosis factor-α (TNF-α), 5’-CGAGTGACAAGCCTGTAGCC-3’ (forward) and 5’GGTGAGGAGCACGATGTCG-3’ (reverse); monocyte chemotactic and activating factor-1 (MCP-1), 5’-TTAACGCCCCACTCACCTGCTG-3’ (forward) and 5’-GCTTCTTTGGGACACCTGCTGC-3’ (reverse); interleukin-6 (IL-6) 5’-CCACTTCACAAGTCGGAGGCTTA-3’ (forward) and 5’-GCAAGTGCATCATCGTTGTTCATAC-3’ (reverse); p22^phox^, 5’-TGGCTACTGCTGGACGTTTCAC-3’ (forward) and 5’-CTCCAGGAGACAGATGAGCACAC-3’ (reverse); p47^phox^, 5’-GTCCCTGCATCCTATCTGGA-3’ (forward) and 5’-GGGACATCTCGTCCTCTTCA-3’ (reverse); p67^phox^, 5’-CAGACCCAAAACCCCAGAAA-3’ (forward) and 5’-AGGGTGAATCCGAAGCTCAA-3’ (reverse); gp91^phox^, 5’-TGGGATCACAGGAATTGTCA-3’ (forward) and 5’-CTTCCAAACTCTCCGCAGTC-3’ (reverse); inducible nitric oxide synthase (iNOS), 5’-GTCACCTACCGCACCCGAG-3’ (forward) and 5’-GCCACTGACACTTCGCACAA-3’ (reverse); endothelial NOS (eNOS), 5’-GGCTCCCTCCTTCCGGCTG-3’ (forward) and 5’-TCCCGCAGCACGCCGAT-3’ (reverse); GAPDH, 5’-ATGTAGGCCATGAGGTCCAC-3’ (forward) and 5’-TGCGACTTCAACAGCAACTC-3’ (reverse).

### Autoradiography of distribution of MKP

^14^C-labeled MKP, Met-[1-^14^C]Lys-Pro (^14^C-MKP), was purchased from GE Healthcare (Buckinghamshire, UK). Radiochemical purity assessed by high-performance liquid chromatography (HPLC) was 98.9%, and its specific radioactivity was 1.85 GBq/mmol. The test solution contained CH-3 (20 mg/ml) and ^14^C-MKP (0.25 mg/ml) dissolved in distilled water, and was prepared just before its administration. SHRs (n = 3) were starved for 16 h, and given the solution orally (5 ml/kg BW, 3.7 MBq/kg). At 15 minutes after administration, the rats were euthanized by isoflurane overdose, frozen in liquid nitrogen, and sliced with a microtome-cryostat (Leica). The sections were dried at −20°C and exposed to imaging plates (Fuji Photo Film Co., Ltd., Tokyo, Japan). After exposure, each autoradiographic image was analyzed using BAS2000 (Fuji Photo Film Co., Ltd.). Blood was collected at 15 min after administration, and radioactivity of the plasma samples was quantified with a liquid scintillation counter (LSC-1000; Aloka, Tokyo, Japan) as reported previously [8].

### Statistical analysis

All values are expressed as mean ± S.D. in the text and figures. Statistical analyses were performed using PASW Statistics for Windows version 17 (SPSS Japan). Comparison of data among the Aβ1-42-injected groups was conducted using one-way ANOVA, and when a significant difference was detected, post hoc analysis was performed with Student’s t test or Welch’s t-test. Values of *p* < 0.05 were considered statistically significant.

## Results

### Effect of CH3 on cognitive function

**Fig 1:**
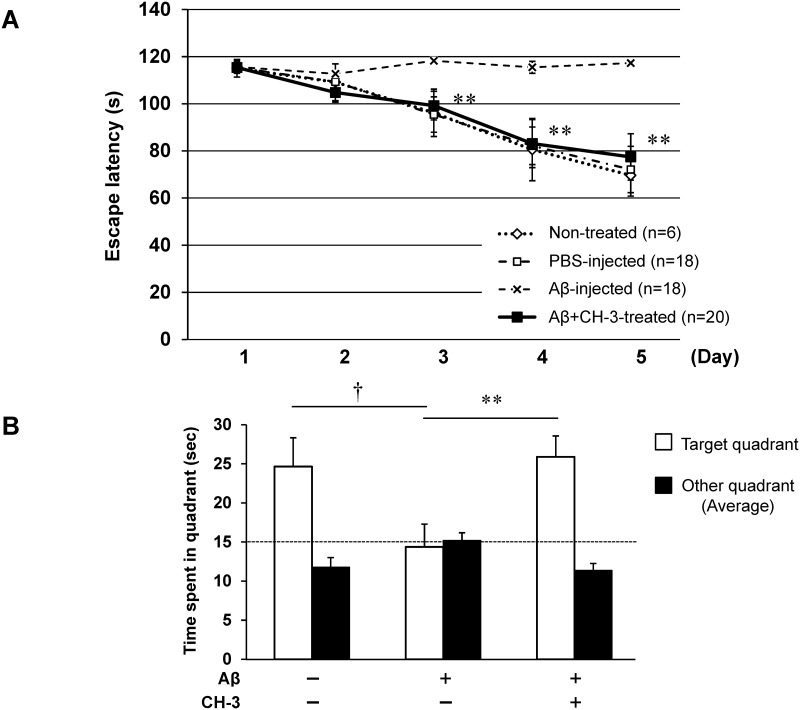
Effect of CH-3 treatment on cognitive function in AD model mice evaluated by Morris water maze test. (**A**) Swim latency in Morris water maze test. (**B**) Time spent in target quadrant including the former platform position. CH-3 was orally administered at 250 mg/kg/day to mice every day starting 2 days before Aβ1–42 injection. n = 18–20 mice in each group. *P<0.05 vs. Aβ1–42 (+) on day 3, day 4 and day 5, respectively. †*P*<0.05 vs. control, **P<0.01 vs. Aβ1–42 (+).

Mice with ICV injection of Aβ1–42 showed significant impairment of spatial learning ability evaluated by the Morris water maze test after 3 weeks, compared with PBS-injected mice (Fig 1A). Daily administration of CH-3 markedly attenuated such cognitive impairment, to the level in PBS-injected mice (Fig 1A). Time spent in the target quadrant including the former platform position was also impaired in Aβ1-42-injected mice compared with control mice; however, Aβ1-42-injected mice with CH-3 treatment exhibited similar ability to recognize the platform compared with PBS-injected mice (Fig 1B). Similarly to previous reports [15], we also confirmed that an ACE inhibitor, perindopril, reduced the cognitive decline in AD mice (S1 Fig).

### Brain inflammation and oxidative stress

**Fig 2:**
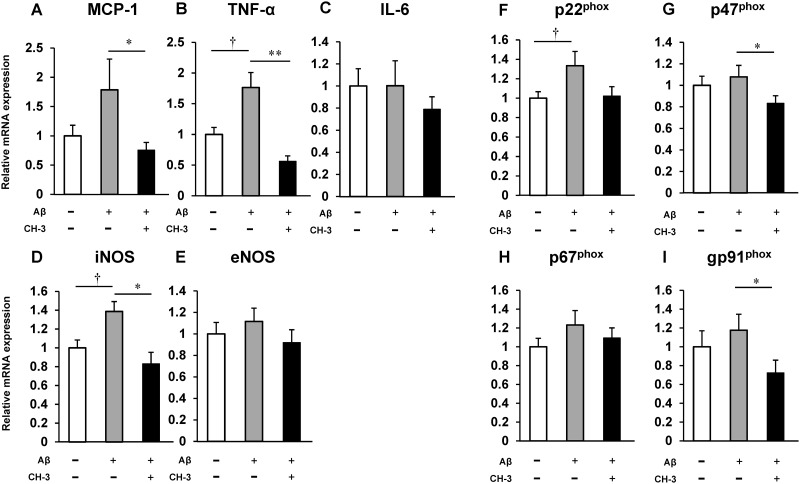
Change in mRNA expression of inflammatory cytokines and NADPH oxidase subunits in hippocampus of AD model mice by CH-3 treatment. mRNA expression of MCP-1 (**A**), TNF-α (**B**), IL-6 (**C**), iNOS (**D**), eNOS (**E**), p22^phox^ (**F**), p47^phox^ (**G**), p67^phox^ (**H**) and gp91^phox^ (I) in hippocampus. n = 8–12 mice in each group. †*P*<0.05 vs. control, *P<0.05 or **P<0.01 vs. Aβ1–42 (+).

To investigate the possible involvement of inflammation and oxidative stress in the improvement of Aβ 1-42-induced cognitive decline by CH-3, the mouse hippocampus was obtained after behavioral testing, and the expression of inflammatory cytokines and NADPH oxidase subunits was assessed by real-time RT-PCR. Aβ1–42 injection significantly enhanced mRNA expression of TNF-α, iNOS and p22^phox^ in the mouse hippocampus compared with PBS injection, and showed a tendency to increase mRNA expression of MCP-1, p47^phox^ and gp91^phox^; whereas treatment with CH-3 markedly reduced Aβ 1-42-induced TNF-α, MCP-1, iNOS, p47^phox^ and gp91^phox^ expression (Fig 2).

### Distribution of MKP after oral administration

**Fig 3:**
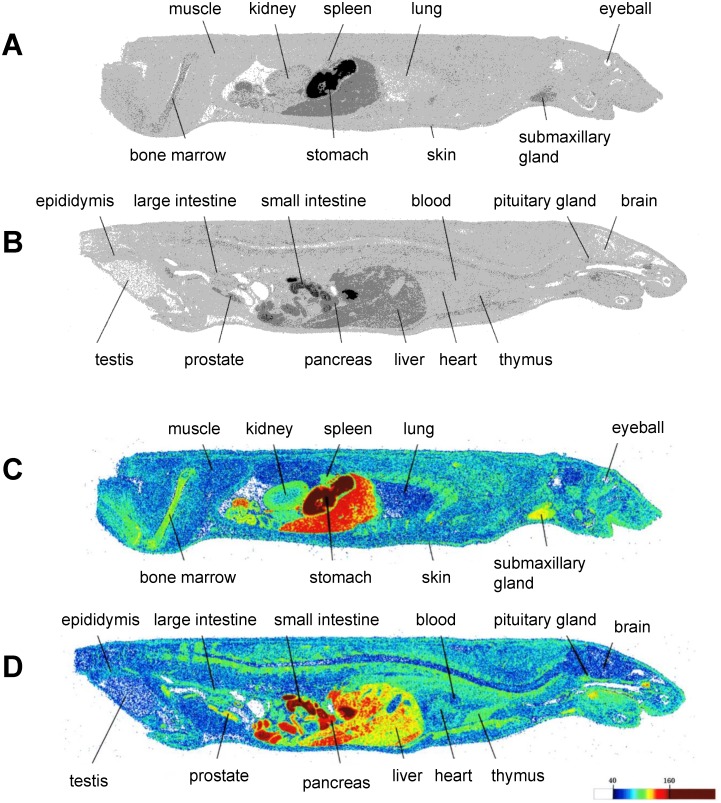
Autoradiographic images of mice after oral administration of MKP labeled with ^14^C. Radioactivity distribution at 15 min after oral administration of ^14^C-MKP and CH-3 to male mice. Dorso-ventral section including kidney (**A**, **C**: colored) and dorso-ventral mesion section (**B**, **D**: colored).

The distribution of radioactivity in tissues was detected by autoradiography after oral administration of ^14^C-MKP. Fig 3 shows the distribution of MKP 15 minutes after oral administration. The administered radioactivity was distributed in many organs and tissues. Of these tissues, radioactivity showed a relatively high concentration in the small intestine, pancreas and liver. It is noteworthy that the signal was also observed in brain tissue. Radioactivity in brain tissue was found to be 87,607 eq·dpm/g and that in plasma samples was 91,182±23,993 dpm/ml (mean±SE; n = 3). These data indicate that orally administered MKP was mainly distributed in the gastrointestinal system, but MKP could penetrate the blood brain barrier (BBB) and enter the brain.

### Effect of MKP on cognitive function

**Fig 4:**
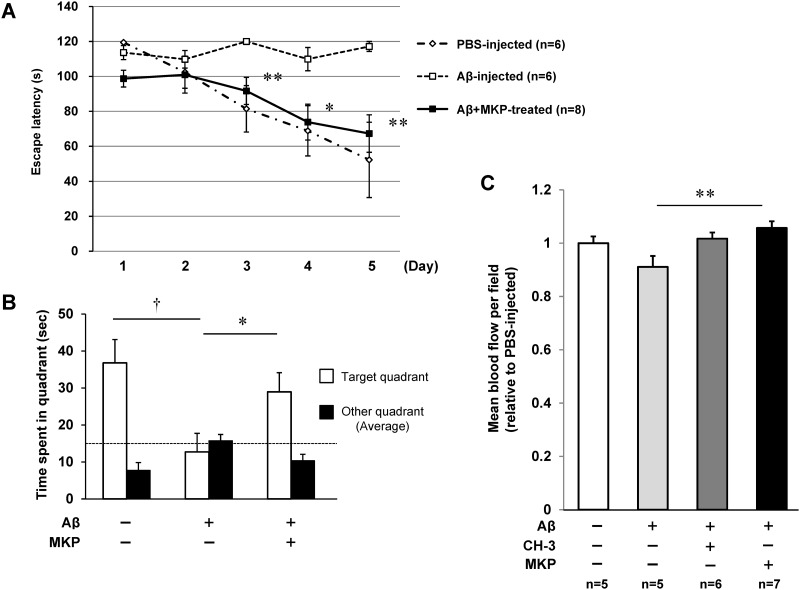
Improvement of cognitive decline by MKP treatment in AD model mice. (**A**) Swim latency in Morris water maze test. (**B**) Time spent in target quadrant including the former platform position in Morris water maze test. (**C**) Cerebral blood flow measured by laser speckle flowmetry after Morris water maze test. MKP was orally administered to mice every day at 0.5 mg/kg/day starting 2 days before Aβ1–42 injection. n = 5–8 mice in each group. †*P*<0.05 vs. control, *P<0.05 or **P<0.01 vs. Aβ1–42 (+).

Finally, we investigated the effect of MKP, which accounts for the main ACE inhibitory effect of CH-3 and has the possibility to penetrate the BBB, on cognitive decline induced by ICV injection of Aβ1–42. Daily treatment with MKP attenuated Aβ 1-42-induced cognitive impairment such as escape latency and time spent in the target quadrant, to the level in PBS-injected mice (Fig 4A and 4B). Interestingly, cerebral blood flow in Aβ1-42-injected mice was significantly reversed by MKP treatment (Fig 4C). However, MKP treatment had no significant effect on cerebral blood flow in PBS-injected mice (S2 Fig). Peptide administration to PBS-injected mice had no effect on cognitive function (S3 Fig). Peptide administration had no effect on blood pressure in both PBS-injected and Aβ-injected mice (S4 Fig and S1 Text).

## Discussion

Here, we focused on bovine casein-derived peptide CH-3, which has a strong ACE inhibitory effect, and demonstrated that administration of CH-3 prevented cognitive decline in AD model mice, with a reduction of inflammation and oxidative stress. In addition, we also confirmed that MKP, a tripeptide in CH-3 that has a strong ACE inhibitory effect with BBB penetration, showed a similar effect to CH-3 and thus may be one of the main players in the prevention of cognitive impairment by CH-3.

AD is the most common form of dementia, accounting for almost two thirds of all cases. Recently, interplay between hypertension and dementia, such as vascular dementia and AD, and neurovascular dysfunction via vascular degeneration induced by chronic hypertension, has been highlighted [22–25]. Epidemiological studies also suggest that midlife hypertension is a risk factor for dementia in the elderly [26]. The renin-angiotensin system (RAS) is a key player in maintenance of blood pressure, and thus, the RAS is a crucial target for preventing dementia. ACE activity is elevated in the brain of AD patients [12], and RAS-modulating medicine has possibility as a new treatment option for AD. In particular, BBB-penetrating RAS inhibitors are thought to have a beneficial effect in suppressing the development of AD. A clinical study, the Perindopril pROtection aGainst REcurrent Stroke Study (PROGRESS), demonstrated that treatment with perindopril, a centrally active ACE inhibitor, reduces the risk of severe cognitive decline and stroke-related dementia [27,28]. Ohrui et al. demonstrated a protective role of long-term use of ACE inhibitors against the development of Alzheimer disease [16]. Moreover, Yamada et al. and Dong et al. also demonstrated preventive effects of perindopril on cognitive impairment in an Alzheimer disease mouse model [15,29]. In fact, we found that perindopril could have a protective effect on AD mouse cognitive function (S1 Fig).

We previously reported that CH-3 has strong ACE inhibitory activity in vitro [8,9]. Orally administered MKP, which contributes 33% of the total ACE-inhibitory activity of CH-3, was rapidly absorbed into plasma, and exhibited an antihypertensive effect in spontaneously hypertensive rats, probably through its ACE inhibitory activity. Because a previous study showed that BBB penetration is crucial for the protective effect of a RAS inhibitor on AD, we measured MKP bioavailability such as absorption and distribution after oral CH-3 administration to determine the fate of MKP, using whole body autoradiography. To analyze MKP bioavailability under the condition in which MKP coexists with many other CH-3-containing peptides, we orally administered a mixed solution of CH-3 and ^14^C-MKP. The autoradiographic images revealed that radioactivity was detected throughout the body 15 min after administration (Fig 3). It is noteworthy that radioactivity was detected in the brain, and its concentrations in brain tissue was 87,607 eq·dpm/g and plasma radioactive intensity was 91,182 dpm/ml. Considering the cerebral blood volume in rats is around 5 ml/100 ml·brain tissue volume [30], the contribution on the signal intensity of brain radioactivity from blood in the cerebral circulation can be estimated to be less than 10%. Additionally, we reported that 15 min after ^14^C-MKP administration, nearly 90% of the radioactivity in plasma was attributable to intact ^14^C-MKP [8]. Therefore, we speculated that the radio-signal detected in the brain was mainly from intact ^14^C-MKP and not merely its metabolite, suggesting the possible ability of MKP to penetrate the BBB.

We hypothesized that CH-3 has a preventive effect on cognitive decline in AD, as have centrally active ACEIs. We investigated this possibility using Aβ1–42 ICV-injected mice, which are commonly used as an animal model of AD [15,19]. ICV infusion of Aβ in the rodent brain can mimic aspects of AD and this model has been used as an AD model mouse by many researchers. Takeda et al. validated the reliability of this model in detail based on three criteria (face validity, construct validity and predictive validity), which suggested that this model is useful to evaluate drugs targeting Aβ and their toxicity, as well as to investigate protective effects of pharmacological modulation of microglial signaling [31]. APP Tg mice, which overexpress APP in the brain, show age-related cognitive decline with Aβ deposition and neuroinflammation and are a useful model of AD. However, APP Tg model mice are based on transgenic overexpression of APP, and an extremely high level of APP might cause some undesirable side effects. For example, overexpression of APP results in increased production of Aβ1–40 and Aβ1–42, but also causes elevated levels of other APP fragments, which may induce an artificial phenotype [32]. When using an ICV injection model, it is possible to administer defined amounts of a specific Aβ species. Aβ1–40 and Aβ1–42 are the major components of senile plaques and are considered to have a causal role in the development and progression of AD. Aβ1–42 is thought to be more toxic than Aβ1–40, and our data confirmed that ICV injection of Aβ1–42 induced cognitive dysfunction evaluated by the Morris water maze test. Interestingly, the cognitive decline was significantly suppressed by daily administration of CH-3 (Fig 1).

Aβ is known to induce cerebral oxidative stress through activation of microglia and astrocytes and to enhance neuroinflammation, leading to neuronal injury and cognitive impairment [33]. This oxidative stress and neuroinflammation are also seen in AD patients. Oxidative damage is observed early in the progression of AD [34]. Paganelli et al. reported that the proinflammatory cytokine TNF-α level is increased in patients with severe AD compared to mild AD [35]. TNF-α in the AD brain is thought to induce overexpression of iNOS (also called NOS2) and peroxynitrite-mediated nitration of protein, leading to nitrosative stress in the AD brain [36]. A variety of agents targeting TNF-α is thought to be a therapeutic strategy for AD. Neuronal oxidative stress and damage seen in the brains of both AD patients and AD model mice are thought to be mainly attributable to microglial nicotinamide adenine dinucleotide phosphate (NADPH) oxidase (NOX) [37]. Therefore, to determine the detailed underlying mechanism of the beneficial effect of CH-3 on Aβ1-42-injected mice, we analyzed the hippocampal expression of TNF-α and NOX subunits. In this study, Aβ1–42 injection significantly enhanced hippocampal TNF-α expression and tended to induce some NOX subunit expression (Fig 2). CH-3 treatment suppressed TNF-α mRNA level and reduced gp91^phox^, p22^phox^ and p47^phox^ levels induced by Aβ injection. Our study also showed that iNOS expression in the brain was reduced to a normal expression level by the administration of CH-3, suggesting that CH-3 could suppress nitrosative stress induced by Aβ. From these data, we supposed that administration of CH-3 prevented cognitive decline in AD models through its suppressive effect on neuroinflammation or oxidative stress.

To verify the relationship between the ACE-inhibitory activity of MKP and the protective effects of CH-3 on AD mice, we administered MKP daily to Aβ-injected mice and evaluated cognitive function. Fig 4 demonstrates that administration of MKP also attenuated the cognitive dysfunction induced by Aβ1–42. We suggest that MKP is the main player in the beneficial activity of CH-3, through its ACE-inhibitory activity.

It is reported that Aβ induced up-regulation of ACE in neuroblastoma cells, and neuronal ACE activity was increased in the brain of both AD model mice and AD patients with Braak stage [12,15], implying that ICV injection of Aβ might induce excess Ang II production in the brain. Ang II in the brain was reported to induce oxidative stress via NADPH oxidase, leading to neuroinflammation, and to inhibit potassium-induced acetylcholine release in slices of rat entorhinal [38] and human temporal cortex [39]. Our data suggested that administration of CH-3 could reduce Ang II induced by Aβ through its ACE inhibitory property (mainly due to MKP), and suppress oxidative stress and neuroinflammation, leading to a preventive effect on cognitive dysfunction in AD model mice. A previous study confirmed that an ACE inhibitor or ARB could enhance cognition in AD model mice without affecting amyloid burden [15, 40]. The authors suggested that the beneficial effect of an ACEI or ARB on AD model mice could be due to suppression of Aβ-induced toxicity through its anti-inflammation and anti-oxidative properties. We assume that the prevention of cognitive impairment by CH-3 and MKP was attributable to the suppression of Aβ-induced toxicity rather than modulation of Aβ deposition. However, the involvement of other mechanisms cannot be excluded, and these should be examined in future studies.

Until now, no clinically successful therapeutic method or drug for the treatment of AD has been reported. In the disease progression of AD, Aβ production and accumulation start around the forties, and oxidative stress and neuroinflammation occur gradually in the brain. It may take more than 20 years for cognitive impairment to manifest. Disease progression is too advanced for treatment by the time AD is diagnosed. Therefore, therapy to reduce Aβ production or suppress oxidative damage and inflammation for several decades would be required. Thus, there is growing interest in identifying safe food ingredients that may serve to prevent AD. Although we didn’t investigate the possibility of an effect of CH-3 on progression of Aβ burden, we revealed its anti-oxidative stress and anti-inflammatory properties. We assumed that CH-3 would be a good candidate, because it has been taken by humans for a long time and is basically safe when administered in moderate doses.

Additional investigations are needed to clarify the effect of CH-3 on AD. First, this AD model utilizes ICV injection of Aβ1–42; therefore, the preventive effect of CH-3 on Aβ deposition in the brain is not well known. Furthermore, it is not well known whether CH-3 could suppress the progression of AD in humans, and how much CH-3 peptide would be needed per day, and it is necessary to compare the mode of treatment to determine whether CH-3 intake or administration of purified MKP would be better.

In conclusion, the present study demonstrated that oral administration of bovine milk peptide, CH-3, to AD model mice not only improved cognitive function but also suppressed the expression of inflammatory cytokines and oxidative stress-related proteins. The suppressive effect of CH-3 is mainly due to the tripeptide MKP. These results suggest therapeutic potential of CH-3 and MKP for preventing cognitive impairment in AD.

## Supporting information

S1 Fig Effect of perindopril on cognitive function of AD model mice evaluated by Morris water maze test. (A) Swim latency in Morris water maze test. (B) Time spent in target quadrant including the former platform position. Perindopril was orally administered at 1 mg/kg/day to mice every day starting 2 days before Aβ1–42 or PBS injection. n = 4–7 mice in each group. *P<0.05 vs. Aβ1–42 (+). †P<0.05 vs. control, **P<0.01 vs. Aβ1–42 (+).
(TIF)

S2 Fig Effect of MKP treatment on cerebral blood flow of PBS-injected mice. Cerebral blood flow was measured by laser speckle flowmetry after Morris water maze test. MKP was orally administered to mice every day at 0.5 mg/kg/day starting 2 days before PBS injection. n = 4–5 mice in each group.
(TIF)

S3 Fig Effect of test reagents on cognitive function of PBS-injected mice. Swim latency in Morris water maze test. CH-3 (250 mg/kg/day) or MKP (0.5 mg/kg/day), perindopril (1 mg/kg/day) was orally administered to mice every day starting 2 days before PBS injection. n = 4–6 mice in each group. The data of PBS-injected group mice (n = 4) are the same as the data of S1 Fig.
(TIF)

S4 Fig Effect of test reagents on blood pressure of AD model mice and PBS-injected mice. Blood pressure was measured by using a noninvasive computerized tail-cuff system. CH-3 (250 mg/kg/day) or MKP (0.5 mg/kg/day), perindopril (1 mg/kg/day) was orally administered to mice every day starting 2 days before Aβ1–42 or PBS injection. n = 4 mice in each group.
(TIF)

S1 Text Supporting method of blood pressure measurement. (PDF)
