# Supplementary figures and images for: Administration of bovine casein-derived peptide prevents cognitive decline in Alzheimer disease model mice
Source: PLoS One. 2017 Feb 3;12(2):e0171515. doi: 10.1371/journal.pone.0171515 (PMC5291428; doi:10.1371/journal.pone.0171515)

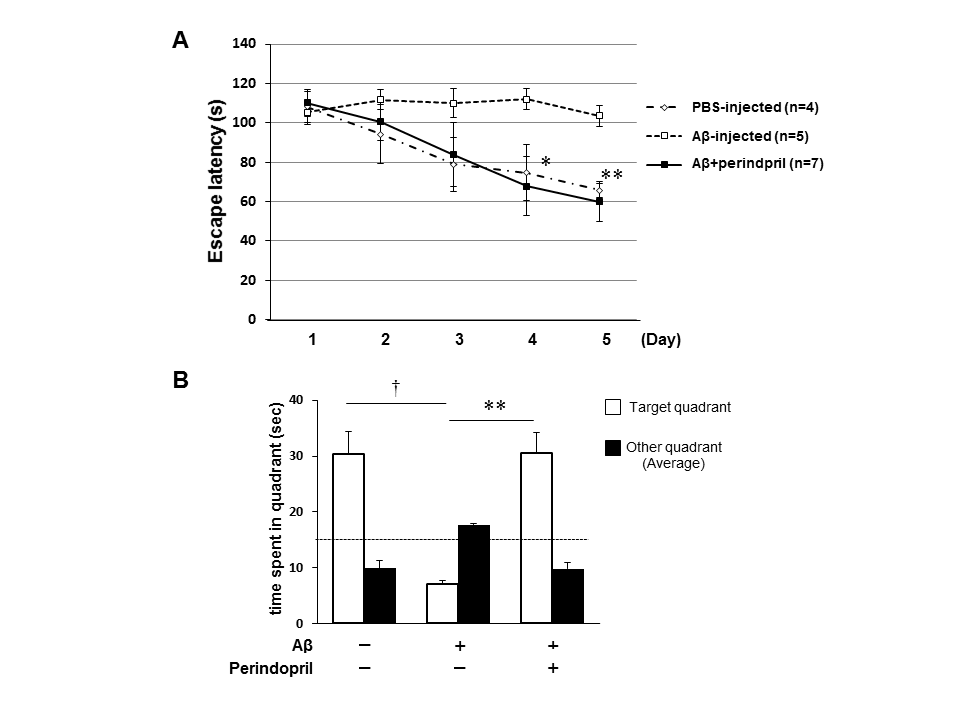

Supplement: S1 Fig — (A) Swim latency in Morris water maze test. (B) Time spent in target quadrant including the former platform position. Perindopril was orally administered at 1 mg/kg/day to mice every day starting 2 days before Aβ1–42 or PBS injection. n = 4–7 mice in each group. *P<0.05 vs. Aβ1–42 (+). †P<0.05 vs. control, **P<0.01 vs. Aβ1–42 (+). (TIF) [file pone.0171515.s001.TIF]

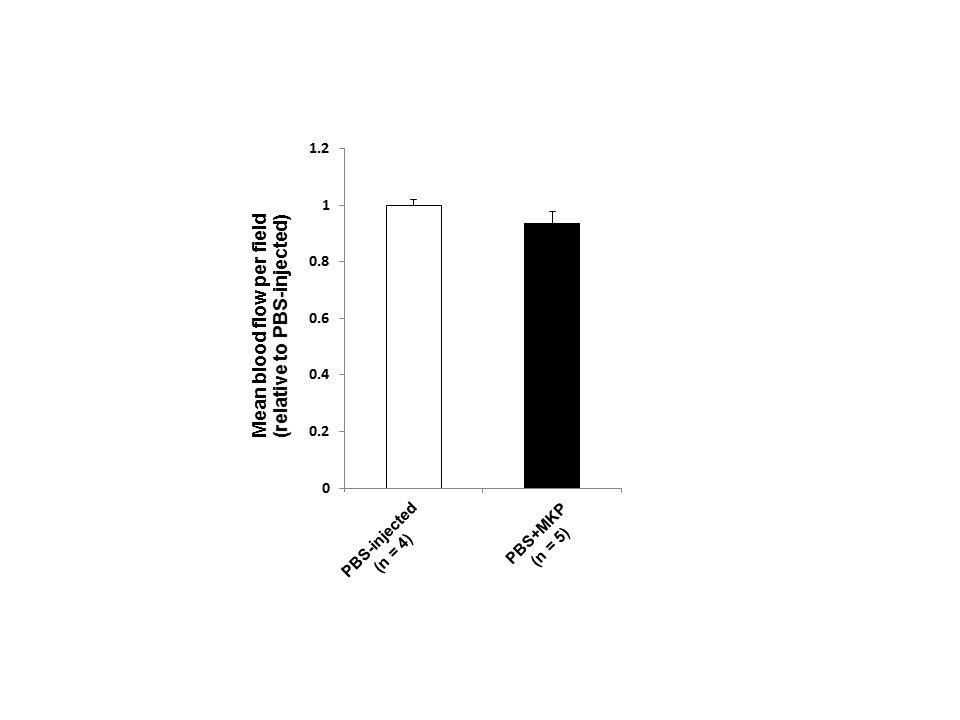

Supplement: S2 Fig — Cerebral blood flow was measured by laser speckle flowmetry after Morris water maze test. MKP was orally administered to mice every day at 0.5 mg/kg/day starting 2 days before PBS injection. n = 4–5 mice in each group. (TIF) [file pone.0171515.s002.TIF]

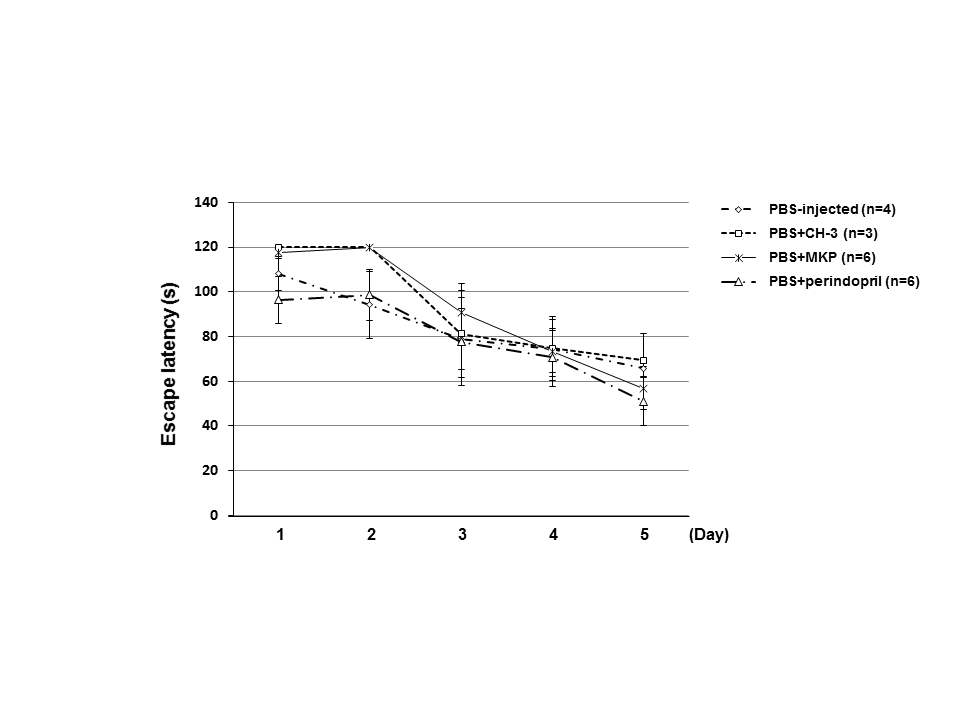

Supplement: S3 Fig — Swim latency in Morris water maze test. CH-3 (250 mg/kg/day) or MKP (0.5 mg/kg/day), perindopril (1 mg/kg/day) was orally administered to mice every day starting 2 days before PBS injection. n = 4–6 mice in each group. The data of PBS-injected group mice (n = 4) are the same as the data of S1 Fig. (TIF) [file pone.0171515.s003.TIF]

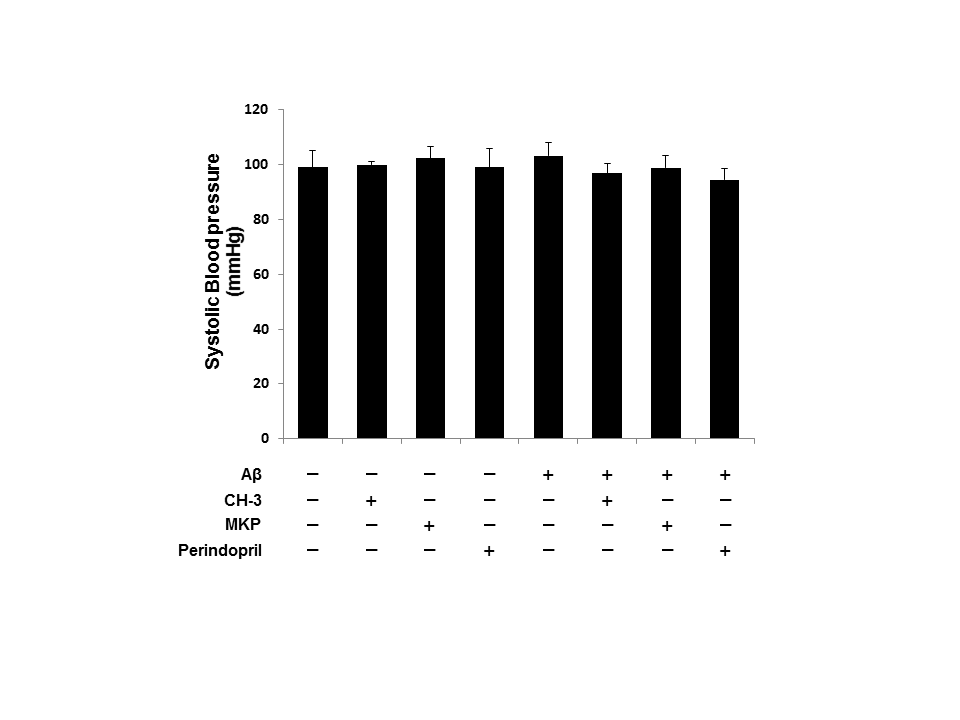

Supplement: S4 Fig — Blood pressure was measured by using a noninvasive computerized tail-cuff system. CH-3 (250 mg/kg/day) or MKP (0.5 mg/kg/day), perindopril (1 mg/kg/day) was orally administered to mice every day starting 2 days before Aβ1–42 or PBS injection. n = 4 mice in each group. (TIF) [file pone.0171515.s004.TIF]
