# Supplementary material for: Administration of bovine casein-derived peptide prevents cognitive decline in Alzheimer disease model mice
Source: PLoS One. 2017 Feb 3;12(2):e0171515. doi: 10.1371/journal.pone.0171515 (PMC5291428; doi:10.1371/journal.pone.0171515)
Supplement: S1 Text — (PDF) [file pone.0171515.s005.pdf]

Supporting Method

Blood Pressure Measurement

We used a noninvasive computerized tail-cuff system to measure blood pressure in conscious mice during reagent administration (BP-98A-L, Softron Co., Ltd., Tokyo, Japan), as described in a previous report [1]. The system was designed to perform all functions automatically, including a programmable routine of cuff inflation and deflation, analysis and assignment of pulse rate and blood pressure, and recording of data electronically. Mice were held in a small mouse pocket on a warming pad thermostatically controlled at 37 °C. Mean systolic blood pressure of fifteen measurements in each group was determined.

Reference

1. Krege JH, Hodgin JB, Hagaman JR, Smithies O (1995) A noninvasive computerized tail-cuff system for measuring blood pressure in mice. Hypertension 25: 1111-1115.
